# Supplementary material for: Type V Collagen Controls Decidual Extracellular Matrix Organization and Angiogenesis During Embryo Implantation
Source: bioRxiv. 2026 Jul 25:2026.07.22.740039. Preprint. [Version 1] doi: 10.64898/2026.07.22.740039 (PMC13419712; doi:10.64898/2026.07.22.740039)
Supplement: Supplement 1 [file media-1.pdf]

Type V Collagen Controls Decidual Extracellular Matrix Organization and Angiogenesis During Embryo Implantation

Mona Gebril[1], Emily Kinneston[1], Rimi Das[1], Joseph Boyd[2], John P Lydon[3], Shanmugasundaram Nallasamy[1]

[1]Department of Obstetrics, Gynecology and Reproductive Sciences, University of Vermont College of Medicine, Burlington, VT, United States. [2]Vermont Integrative Genomics Resource, University of Vermont, Burlington, VT, United States. [3]Department of Molecular and Cellular Biology, Baylor College of Medicine, Houston, TX 77030, United States.

SUPPLEMENTARY METHODS

Superovulation

Three-week-old *Col5a1*$^{d/d}$ and *Col5a1*$^{f/f}$ mice were injected intraperitoneally with 5 IU pregnant mare serum gonadotropin (PMSG; Sigma-Aldrich). After 48 h, mice received 5 IU human chorionic gonadotropin (hCG; Sigma-Aldrich). At 16–18 h after hCG injection, mice were euthanized, and oocytes were collected from the oviductal ampulla and counted.

Measurement of serum estradiol and progesterone levels

Serum samples were collected on GD6 from *Col5a1*$^{f/f}$ and *Col5a1*$^{d/d}$ mice and submitted to the Ligand Assay and Analysis Core of the Center for Research in Reproduction at the University of Virginia for measurement of circulating 17β-estradiol (E2) and progesterone (P4) concentrations.

RNAscope

Paraffin-embedded tissues were sectioned, deparaffinized in xylene, and rehydrated in 100% ethanol. Endogenous peroxidase activity was blocked with RNAscope Hydrogen Peroxide (Cat. No. 322330, Advanced Cell Diagnostics) for 10 min at room temperature, followed by washing in distilled water. Target retrieval was performed using 1× target retrieval solution (Cat. No. 322000, Advanced Cell Diagnostics) for 15 min, followed by washes in distilled water and 100% ethanol. A hydrophobic barrier was drawn around each section, and slides were incubated with Protease Plus (Cat. No. 322330, Advanced Cell Diagnostics; 5 drops per slide) in a HybEZ oven (Boekel Scientific) at 40 °C for 30 min. Sections were then hybridized with a *Col5a1* probe (Cat. No. 521291, Advanced Cell Diagnostics; 4 drops per slide) at 40 °C for 2 h. Signal amplification was carried out using Amp 1–6 reagents (Cat. No. 322310, Advanced Cell Diagnostics) according to the manufacturer’s protocol. Signals were detected using an equal mixture of BROWN-A and BROWN-B reagents (Cat. No. 322310, Advanced Cell Diagnostics). Sections were counterstained with hematoxylin for 2 min, washed in distilled water, dehydrated through 70% and 95% ethanol followed by xylene, and mounted with VectaMount (Vector Laboratories). Whole uterine sections were captured using the Leica VERSA8 whole slide imager and subsequently processed using Aperio ImageScope and ImageJ software.

SUPPLEMENTARY FIGURES

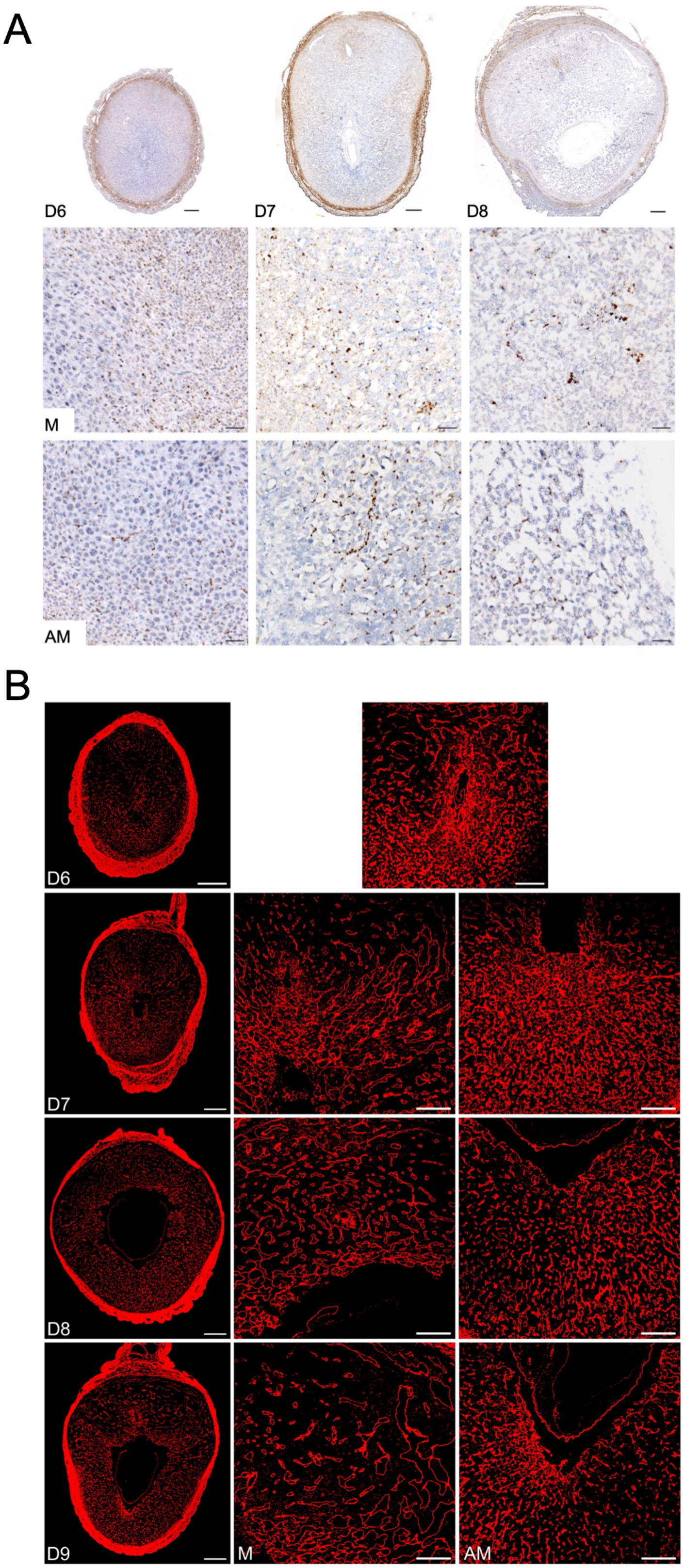

Supplementary Fig. 1. Localization of *Col5a1* in the mouse uterus.
(A) Localization of *Col5a1* mRNA in uterine sections from GD6 to GD8 mice using RNAscope (n = 3 per gestational time point). Scale bar: 250 µm (top panel) and 50 µm (middle and lower panels). Top panel: panoramic view of whole uterine sections; AM, images captured from the antimesometrial region; M, images captured from the mesometrial region.
(B) Confocal imaging of COL5A1 in frozen mouse uterine cross-sections from GD6 through GD9. Left panel: panoramic view of whole uterine sections; AM, images captured from the antimesometrial region; M, images captured from the mesometrial region. Imaging settings were adjusted for each individual image to optimize visualization of morphology. Representative images from three independent experiments. Scale bar: 500 µm (left panel) and 200 µm (all other images).

A

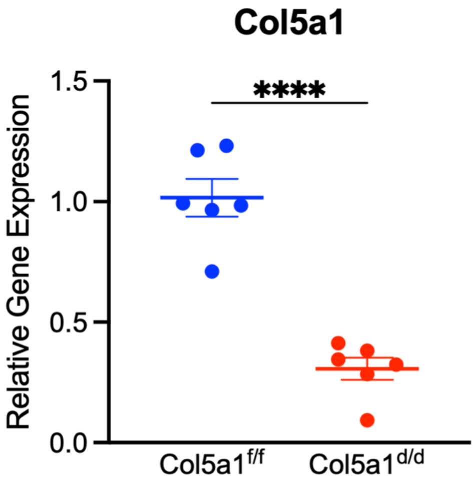

B

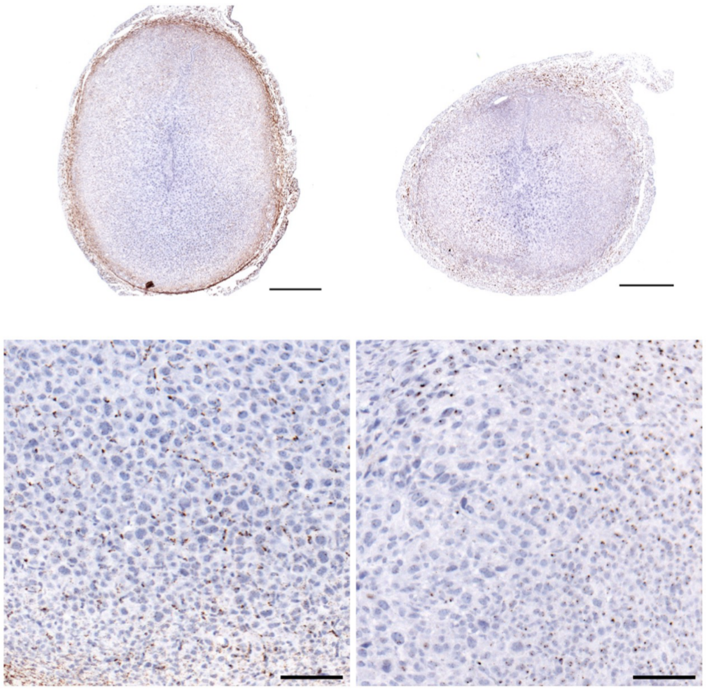

Supplementary Fig. 2. Assessment of the efficiency of *Col5a1* deletion in the mouse decidua.
(A) qPCR analysis of *Col5a1* gene expression in GD6 decidua from *Col5a1*$^{f/f}$ and *Col5a1*$^{d/d}$ mice (n = 6 per genotype; ****P < 0.0001).
(B) Localization of *Col5a1* mRNA in uterine sections from GD6 *Col5a1*$^{f/f}$ and *Col5a1*$^{d/d}$ mice (n = 3 per gestational time point). Scale bar: 500 µm (top panel) and 100 µm (lower panel).

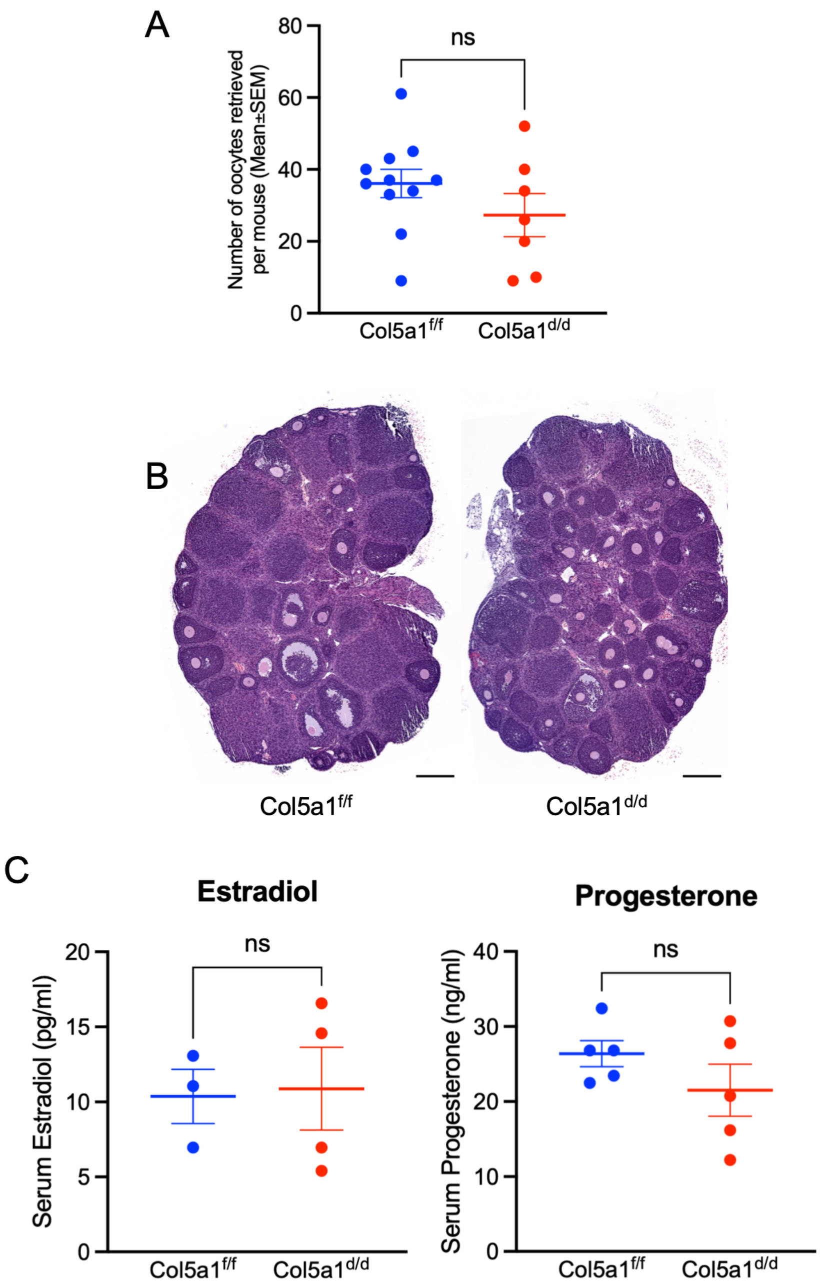

Supplementary Fig. 3. Ovarian function is preserved in *Col5a1$^{d/d}$* mice.
(A) Dot plot showing the number of oocytes retrieved from the oviduct following superovulation, demonstrating comparable counts between groups (*Col5a1$^{f/f}$*: n = 11; *Col5a1$^{d/d}$*: n = 7). ns, not significant.
(B) Histological evaluation of ovaries from *Col5a1$^{f/f}$* and *Col5a1$^{d/d}$* mice following the superovulation procedure (n = 3 mice per genotype). Scale bar: 100 µm.
(C) Measurement of serum estradiol and progesterone levels at GD6 in *Col5a1$^{f/f}$* and *Col5a1$^{d/d}$* mice (n = 3–5 mice). ns, not significant.

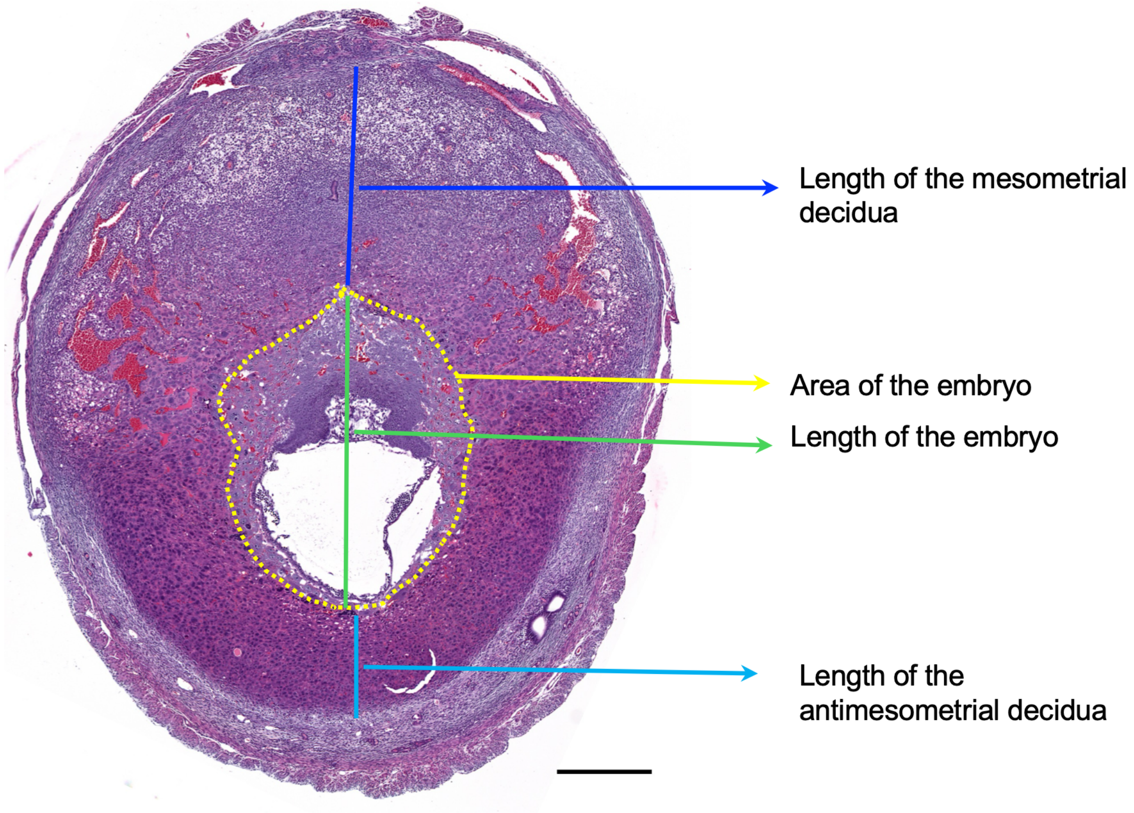

Supplementary Fig. 4. Measurement guide for the decidua and embryo.
Uterine sections were prepared from *Col5a1*$^{f/f}$ and *Col5a1*$^{d/d}$ mice at GD6–GD8 and stained with hematoxylin and eosin. Measurements, including the lengths of mesometrial and antimesometrial decidual regions, as well as embryo length and area, were obtained as outlined in the provided image using Aperio ImageScope software.

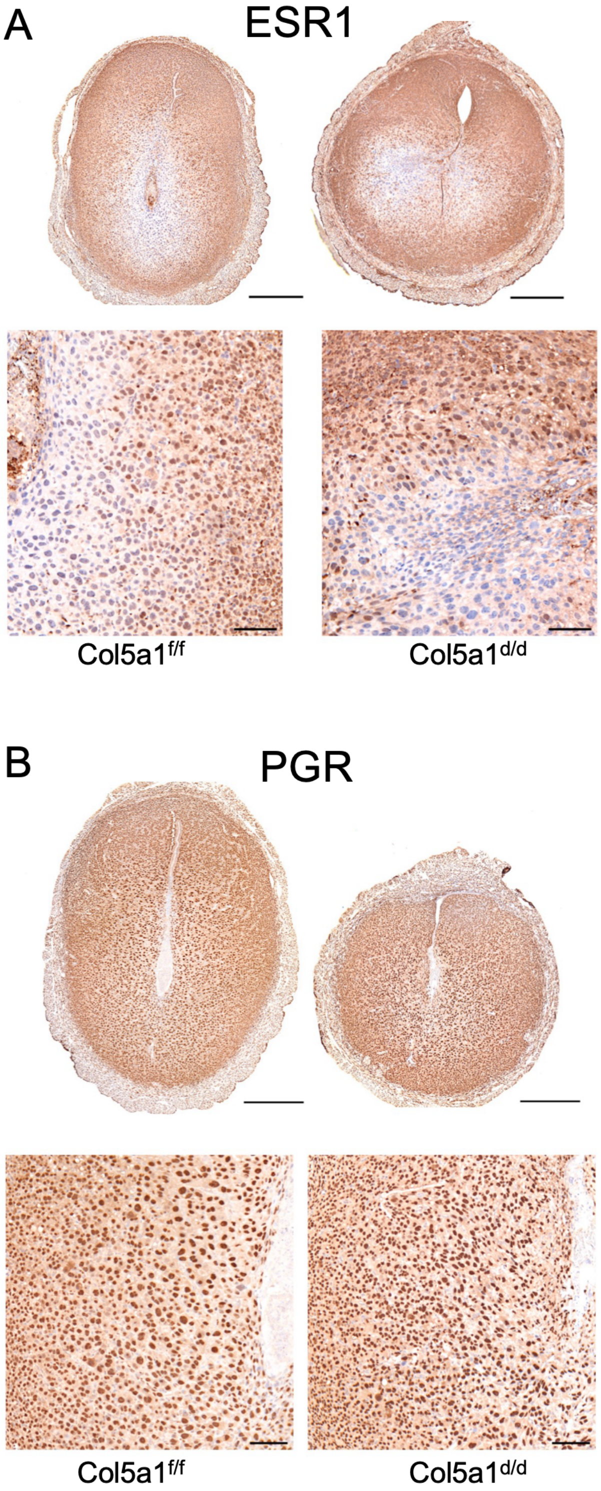

Supplementary Fig. 5. Uterine ESR1 and PGR expression are not affected in *Col5a1*$^{d/d}$ mice.
(A) Immunohistochemical localization of ESR1 in GD6 uterine sections from *Col5a1*$^{f/f}$ and *Col5a1*$^{d/d}$ mice.
(B) Immunohistochemical localization of PGR in GD6 uterine sections from *Col5a1*$^{f/f}$ and *Col5a1*$^{d/d}$ mice.
Scale bar: 500 µm (top panel) and 100 µm (lower panel).

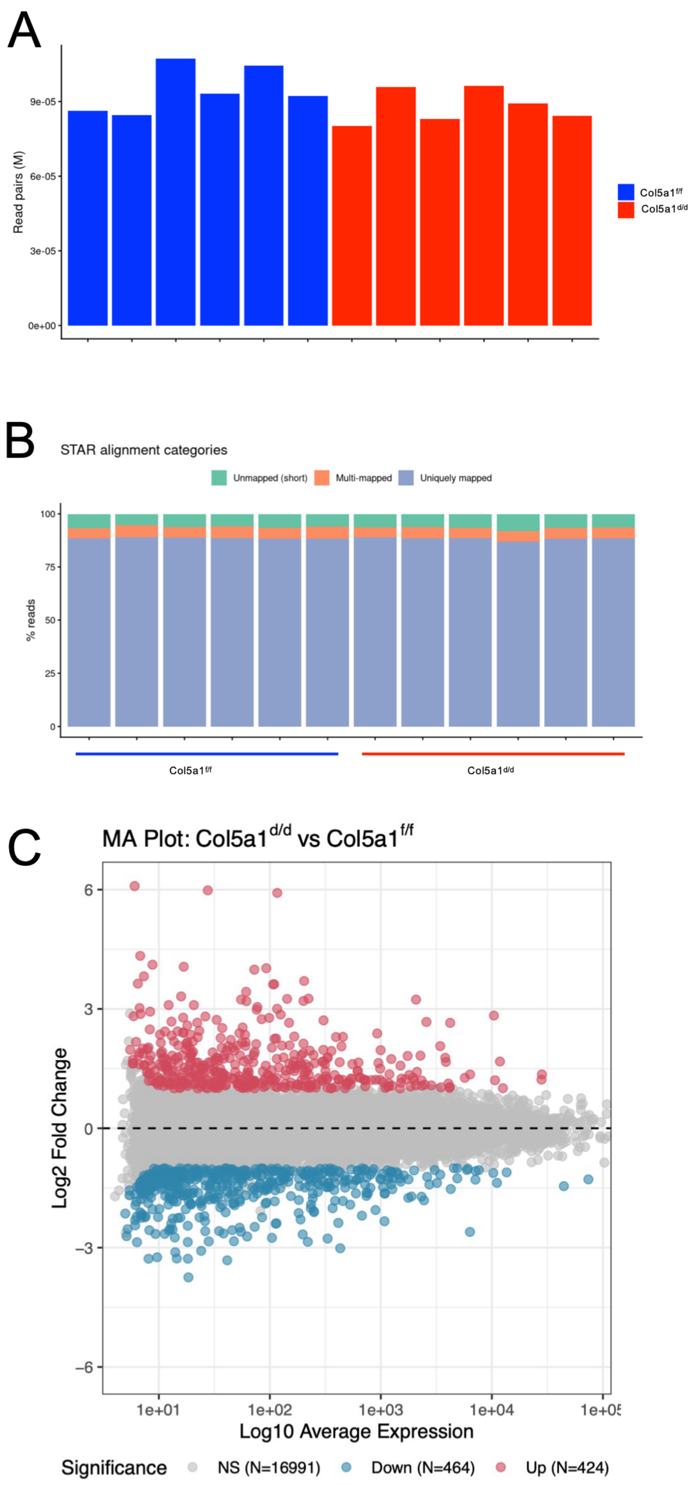

Supplementary Fig. 6. Quality-control metrics from the nf-core/rnaseq pipeline.
(A) Graph showing total read counts per sample generated by FastQC.
(B) Graph showing STAR alignment statistics for reads mapped to the reference genome, categorized as unmapped, multi-mapped, and uniquely mapped reads.
(C) MA plot showing log fold change relative to average abundance, where points distant from the horizontal center line indicate stronger condition-associated expression changes.

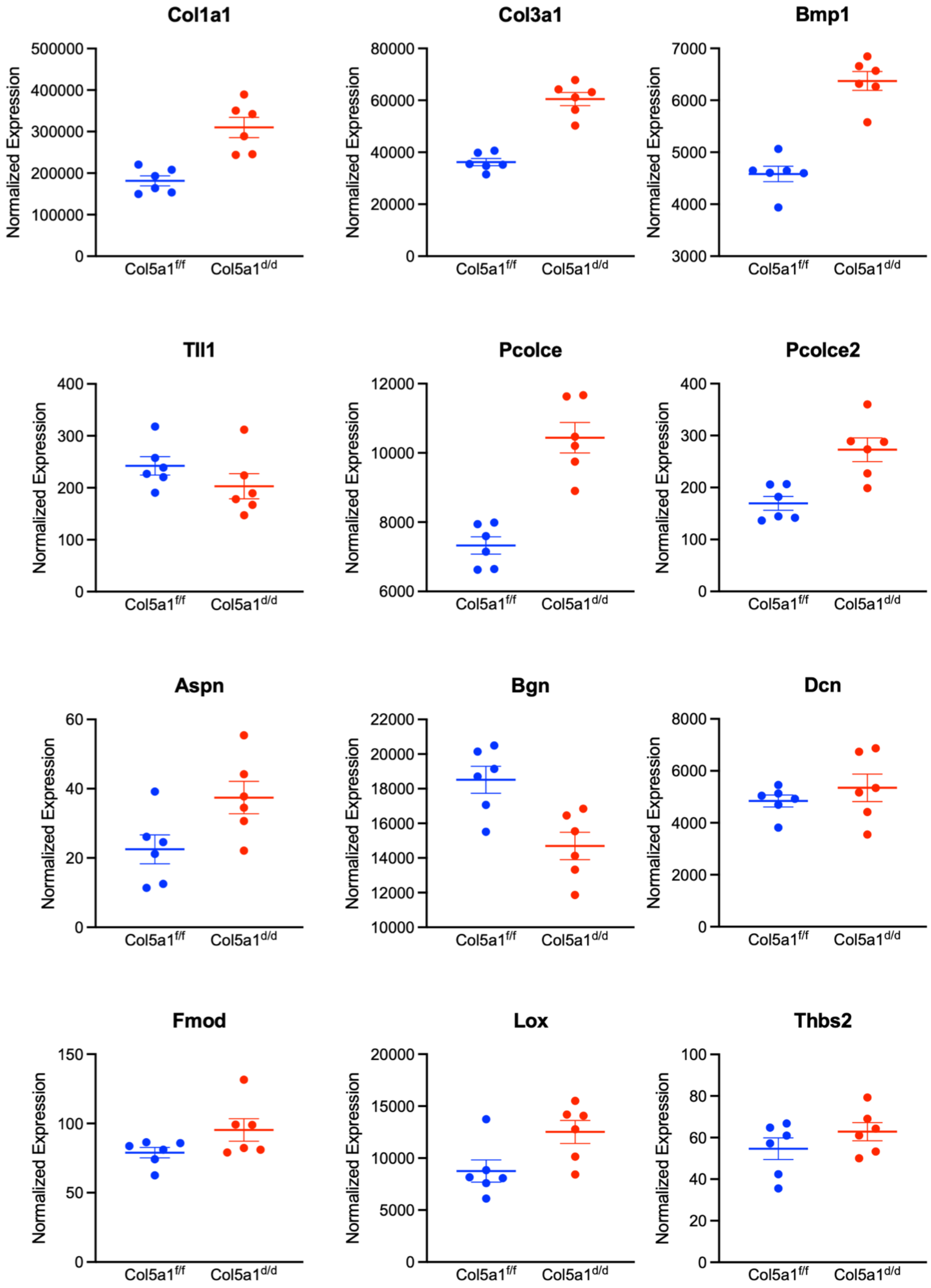

Supplementary Fig. 7. Gene expression of factors involved in fibrillar collagen synthesis, processing, and assembly that are not altered in *Col5a1$^{d/d}$* decidua.

Normalized expression of genes in *Col5a1$^{f/f}$* and *Col5a1$^{d/d}$* decidua, as identified by RNA sequencing analysis. Expression of these genes is not significantly altered based on both thresholds ($|\log_2$ fold change$| > 1$ and adjusted $P < 0.05$).

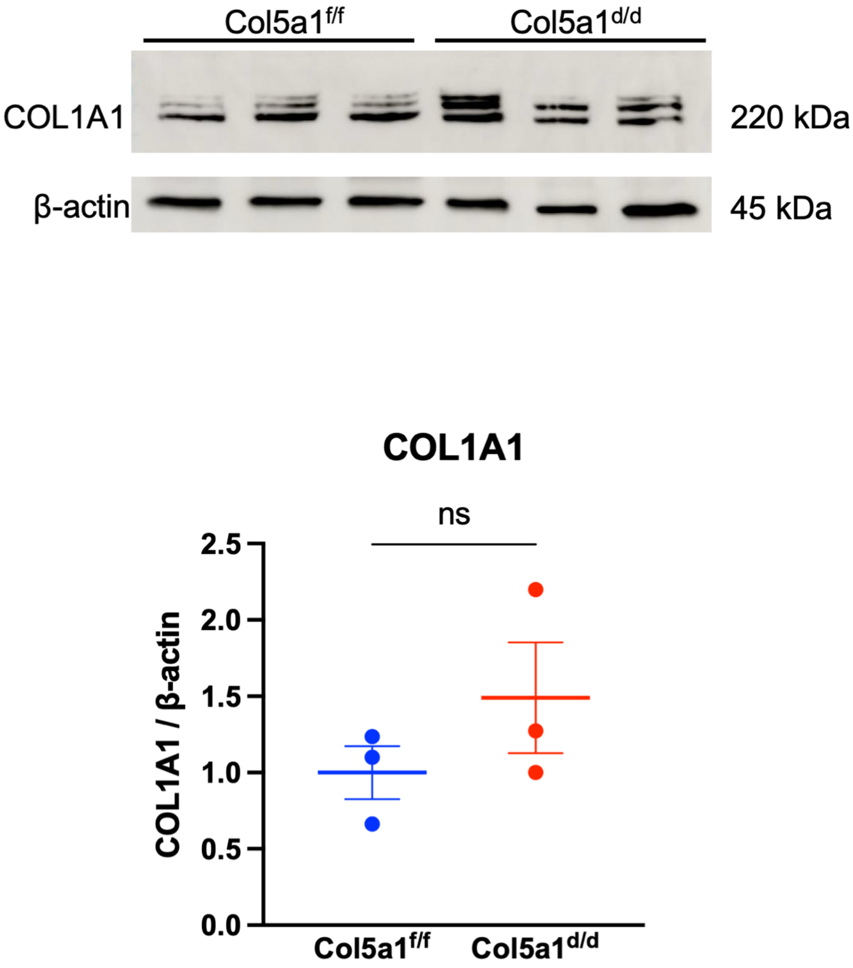

Supplementary Fig. 8. COL1A1 production is not affected in *Col5a1*-deficient decidua. Western blot analysis of COL1A1 protein levels in GD6 decidua from *Col5a1*$^{f/f}$ and *Col5a1*$^{d/d}$ mice. Quantification of COL1A1 protein levels is shown in the graph.

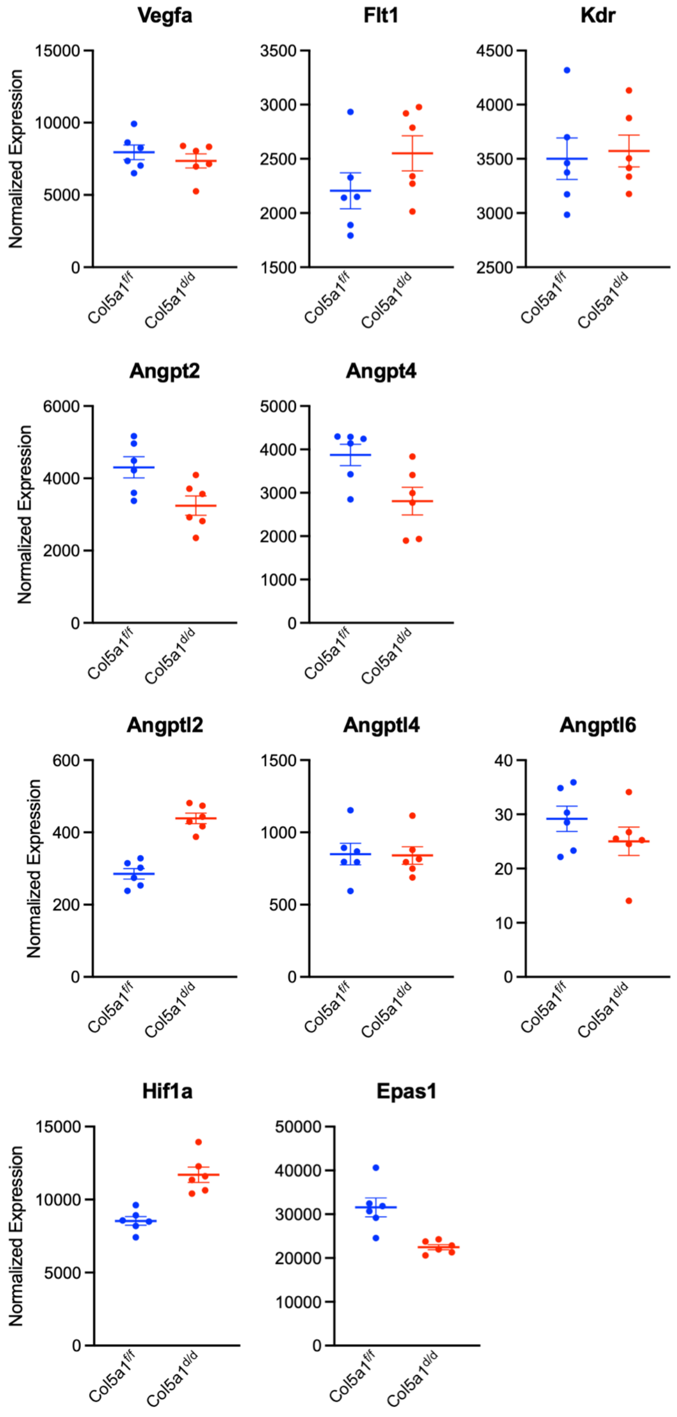

Supplementary Fig. 9. Decidual angiogenesis markers that remain unaltered in *Col5a1*-deficient decidua.

Normalized expression of genes in $Col5a1^{f/f}$ and $Col5a1^{d/d}$ decidua, as identified by RNA sequencing analysis. Expression of these genes is not significantly altered based on both thresholds (|$\log_2$ fold change| > 1 and adjusted P < 0.05).

SUPPLEMENTARY TABLES

Supplementary table 1. Six months breeding experiment to evaluate reproductive phenotype.

Genotype	No. of mice	No. of litters weaned	No. of litters per mouse (Mean ± SEM)	No. of pups born	No. of pups per litter (Mean ± SEM)
$Col5a1^{f/f}$	7	37	5.3 ± 0.2	250	6.8 ± 0.4
$Col5a1^{d/d}$	6	0	0	0	0

Supplementary table 2. The total number of number of uteri examined per genotype with their total number of healthy and resorbed implantation sites counted at each gestational time point from $Col5a1^{f/f}$ and $Col5a1^{d/d}$ mice.

	**$Col5a1^{f/f}$**			**$Col5a1^{d/d}$**		
	Total number of uteri examined	Total number of healthy implantation sites	Total number of resorbed implantation sites	Total number of uteri examined	Total number of healthy implantation sites	Total number of resorbed implantation sites
**Gestation Day 6**	17	128	0	18	129	0
**Gestation Day 7**	15	115	0	21	145	0
**Gestation Day 8**	17	129	0	25	136	28
**Gestation Day 9**	6	41	0	10	10	54
**Gestation Day 10**	4	33	0	4	0	30
**Gestation Day 12**	3	20	0	3	0	20

Supplementary table 3. The list of primers used in this study

Oligo name	Forward	Reverse
**Col5a1**	GCTGAAAATACACACAAAGTAAACAGAA	TGTACCGTTTGGCCTGGAAT
**Prl8a2**	AAACCCACCAGCTCATGGAC	GGAGTGATCCATGCACCCAT
**Bmp2**	CAAAGCAGGACCAGTGGGAA	AGCCCCCTGGAAGGGATTAT
**Wnt4**	GTACCTGGCCAAGCTGTCAT	CCGGAACTGGTATTGGCACT
**Gja1**	TGGACAAGGTCCAAGCCTACTC	TCCCCAGGAGCAGGATTC T
**Rplpo**	CACTGGTCTAGGACCCGAGAAG	GGTGCCTCTGGAGATTTTCG
